# Stigmatization of people living with HIV/AIDS by healthcare workers at a tertiary hospital in KwaZulu-Natal, South Africa: a cross-sectional descriptive study

**DOI:** 10.1186/1472-6939-14-S1-S6

**Published:** 2013-12-19

**Authors:** Temitayo O Famoroti, Lucy Fernandes, Sylvester C Chima

**Affiliations:** 1 Charles James Hospital, Department of Health, Province of KwaZulu-Natal, Durban South Africa; 2 Department of Public Health, School of Health Care Sciences, University of Limpopo, MEDUNSA campus, South Africa; 3 Programme of Bio & Research Ethics and Medical Law, Nelson R Mandela School of Medicine & School of Nursing and Public Health, College of Health Sciences, University of KwaZulu-Natal, Durban, South Africa

**Keywords:** HIV/AIDS, Africa, Attitude, Comfort, Discrimination, Healthcare workers, PLWHA, Stigmatization

## Abstract

**Background:**

The issue of stigma is very important in the battle against HIV/AIDS in Africa since it may affect patient attendance at healthcare centres for obtaining antiretroviral (ARV) medications and regular medical check-ups. Stigmatization creates an unnecessary culture of secrecy and silence based on ignorance and fear of victimization. This study was designed to determine if there is external stigmatization of people living with HIV and AIDS (PLWHA) by health care workers (HCWs) at a tertiary hospital in KwaZulu-Natal (KZN) province, South Africa. The study investigated the impact of knowledge of HIV/AIDS by HCWs on treatment of patients, as well as the comfort level and attitude of HCWs when rendering care to PLWHA.

**Methods:**

A descriptive cross sectional study was designed to collect data using an anonymous self-administered structured questionnaire from 334 HCWs. The study was conducted in clinical departments of a large multidisciplinary 922-bed tertiary care teaching hospital in Durban, KZN.

**Results:**

Overall HCWs had an above average knowledge about HIV/AIDS although some gaps in knowledge were identified. Tests of statistical significance showed that there was association between level of education and knowledge of HIV/AIDs (p ≤ 0.001); occupation and knowledge of HIV/AIDS (p ≤ 0.001); and gender and knowledge of HIV/AIDS (p = 0.004). Test for comfort level was only significant for gender, with males showing more comfort and empathy when dealing with PLWHA (p = 0.003). The study also revealed that patients were sometimes tested for HIV without informed consent before surgery, due to fear of being infected, and there was some gossiping about patients' HIV status by HCWs, thereby compromising patient confidentiality. The majority of HCWs showed a willingness to report incidents of stigmatization and discrimination to higher authorities, for better monitoring and control.

**Conclusions:**

Although knowledge, attitude and comfort level of HCWs taking care of PLWHA was above average, enforcement of existing antidiscrimination laws and continuing education in medical ethics and healthcare law, would greatly improve the performance of HCWs taking care of PLWHAs. More psychological support and counselling should be provided to HCWs, to further reduce the impact of stigmatization and discrimination against PLWHA.

## Background

Acquired immune deficiency syndrome (AIDS) was first identified in the 1980's and since then has spread globally causing one of the most dreaded pandemics of modern times [1]. The virus affects the CD4 cells that help in fighting against diseases and infections in humans. HIV infection predisposes to a host of life threatening opportunistic infections, tumours and diseases such as pulmonary tuberculosis, Cryptococcal meningitis, Kaposi's sarcoma, progressive multifocal leukoencephalopathy and many more [2]. HIV infection is spread primarily through sexual contact with an infected person during unprotected sex and in other ways including sharing of needles with someone who is infected. The infection can also be contracted by transfusions of infected blood or blood components. In addition babies born to HIV-infected women may become infected during pregnancy or through breast-feeding [3]. Currently, there is no known cure for HIV/AIDS although current treatment with antiretroviral drugs (ARVs) has led to an increase in life expectancy and improved quality of life following development and use of ARVs [4]. The availability of ARVs has changed the course of HIV/AIDS from a rapidly fatal disease to a chronic and more manageable disease which invariably improves quality of life of people living with HIV/AIDS (PLWHA) [5].

### HIV/AIDS in South Africa

Sub-Saharan Africa is home to about 10% of the global population, but reports suggest 60% of all people living with HIV/AIDS globally live in sub-Saharan Africa [6]. Southern Africa has been described as the epicentre of the global epidemic with countries in the region registering some of the highest HIV/AIDS prevalence rates in the world. The Republic of South Africa is severely affected with an estimated 5.7 million persons living with HIV in 2009. South Africa's HIV epidemic is hyper-endemic due to high rate of HIV prevalence [7-9] and based on currently available data the national prevalence rate is about 10% [10]. The modes of HIV transmission in sub-Saharan Africa (SSA) is predominantly heterosexual followed by mother to child transmission. Other drivers of HIV transmission in SSA include migration, low perceptions of risk and multiple concurrent sexual partnerships [3]. The high incidence of HIV/AIDs in South Africa has been reportedly associated with low HCW motivation and attrition from work especially among nurses [7].

### Stigmatization and discrimination in HIV/AIDS

Stigmatization and discrimination against PLWHA has been one of the hallmarks of the global HIV/AIDs pandemic [1,11]. Stigma may be defined as any attribute that is deeply discrediting and results in the reduction of a person or group "from a whole and usual person to a tainted, discounted one" [12]. Stigmatization could lead to delays and failures in seeking treatment by PLWHA and delays in diagnosis of high risk patients [13-15]. This may contribute towards continuous spread of the disease within the community, impact on healthcare services in general and derail the curtailment of the global HIV-AIDS pandemic [14-16]. Discrimination may be described as a form of "enacted stigma" or the negative acts that result from stigma which serve to devalue and reduce the life chances of the stigmatized. Such discriminatory acts against PWLHA could lead to denial of rights to health, education, and employment [11]. This is evidenced by legal cases such as the Nigerian case of *Georgina Ahamefule v. Imperial Medical Centre *[17]. Here, a HCW with suspected AIDs-related opportunistic infection was tested for HIV without informed consent, and without counselling. When she tested positive, she was promptly fired by the employer. She subsequently suffered severe psychological and emotional trauma and miscarriage of pregnancy. The patient claimed "humiliation, stigmatization and discrimination" because of the doctors refusal to offer her appropriate treatment following her miscarriage because of her HIV status. In judgment, Idowu J of the Lagos High Court held that the termination of the HCW's employment was illegal, unlawful, and based on malice and bad faith. The Court also held that the employer's action in subjecting the claimant to HIV testing without informed consent constituted unlawful battery, and that not affording the claimant pre or post-test counselling for HIV testing constituted professional negligence. Finally, denying the claimant medical care on grounds of her HIV-positive status constituted violation of the right to health guaranteed under article 16 of the African Charter on Human and People's Rights [18] and the international covenant on economic, social and cultural rights [19]. Similarly, in the South African case of *Jansen van Vuuren v. Kruger *(1993), the Supreme Court of Appeal held that disclosure of a patient's HIV status without consent constituted a breach of doctor-patient confidentiality [20]. In this case a doctor diagnosed a patient with HIV and shared this information with other medical colleagues, not involved in the patient's care, while playing a game of golf-a behaviour which may be described as 'gossip'. It has been averred that stigmatization can be overt and may constitute libel, slander, or defamation of persons who are stigmatized [21]. From the foregoing it is evident that the practice of stigmatization and discrimination of PLWHA by HCWs has been occurring in healthcare practice in Africa and elsewhere since the advent of the HIV/AIDs pandemic. It has also been suggested that reduction of HIV/AIDS-related stigma and discrimination amongst HCW would be helpful not only for PLWHA, but also for healthcare professionals who often show reluctance and delay in accessing healthcare services because of the fear of stigma and discrimination [22]. There have been several previous studies on stigmatization and discrimination of PLWHA by HCWs globally [23]. A Nigerian study found that 90.5% of HCWs had previous HIV/AIDS training and the overall mean knowledge among these physicians was interpreted as moderate [24]. However, the mean attitude score amongst this cohort was 110.6, interpreted as an indication that this cohort of physicians harboured some negative attitude towards patients with HIV/AIDS. This finding of a negative attitude towards PWLHA was confirmed by the fact that 95.3% of the participants had also previously refused to provide care for HIV positive patients [24]. By contrast, a Chinese study reported that HCWs in China who had more HIV related training tend to show significantly lower prejudicial attitudes towards PLWHA [25]. The same study indicated that when compared to nurses, doctors were more willing to have social interactions with HIV/AIDS patients, i.e. strike up a conversation, attend the same party or work in the same office [25]. Another study from Poland comparing the education level of HCWs reported that doctors with a comprehensive HIV education showed less prejudice and to a lesser extent did not support mandatory HIV testing of patients without consent when compared to their counterparts with a lesser amount of training [26]. The same phenomenon was observed amongst Polish nurses where the trained nurses also showed less prejudice when compared to their colleagues with less training [26]. Similarly an Indian study [27] concluded that HCWs with more knowledge regarding HIV/AIDS showed less prejudice to PLWHA and that doctors were less prejudiced when compared to nurses in their attitude towards PWLHA. This study also reported that 68% of the HCWs were judgmental in that they believed that HIV is spread due to immoral behaviour such as promiscuity, extramarital affairs and drug abuse [27]. A total of 90% of HCWs who participated in the Indian study endorsed the practice of conducting mandatory HIV testing prior to surgery and 61% disagreed with the requirement to seek patient's informed consent prior to HIV testing [27]. This study also reported that in certain cases confidentiality is breached where beds are labelled as "danger" for HIV positive patients; and it was also found to be a common practice for nurses to wear gloves for casual contacts, like giving medication when dealing with HIV positive patients [27]. According to reported literature some of the reasons for the negative attitude of health care providers towards HIV-positive patients are fear of contagion [28], while other studies have reported that HCWs are not always knowledgeable about appropriate procedures for maintaining patient confidentiality [29]. External stigma and discrimination against PLWHA also manifests as mandatory testing of patients for HIV before surgery or during delivery of women in labour, unnecessary use of protective gear even for routine examinations and casual contact, although this may be due to regulations requiring HCWs to use universal precautions. Breach of confidentiality manifests as disclosure of patients HIV status to third parties without consent, or discussing the illness of PLWHA with other hospital staff in a demeaning manner. All of these circumstances can affect the way the infected person see themselves (internal stigma) [30]. As a result, stigmatized individuals or groups may accept that they "deserve" to be treated poorly and unequally, making resistance to stigma and resulting discrimination even more difficult [12]. Stigmatization could also be externalized (discrimination) leading to exclusion by family and community, loss of respect, physical and verbal abuse or violence, humiliation and termination of employment [11,17,20]. The current study was designed to identify any factors that contribute to stigmatization and discrimination of PLWHA by HCWs at a tertiary hospital in KwaZulu-Natal province, South Africa.

## Methods

The rationale for this study was based on reports in the scientific literature on stigmatization of HIV positive patients and the consequences of stigmatization and discrimination on the health seeking behaviour of HIV/AIDS patients [12-16]. Therefore we wanted to determine if there are practices by HCWs at local hospitals that could be perceived as stigmatization by PLWHA. Such alleged behaviour by HCWs could spill over into a delay in testing of individuals who are at high risk of being infected with HIV [13], thereby leading to increased transmission of HIV within the community [14]. It could also lead to the reluctance of those known to be HIV positive or PWLHA to seek medication and treatment [15]. The main aim of the study was to determine the level of knowledge, attitude and comfort of HCWs regarding HIV/AIDS, and its influence on rendering healthcare services to HIV/AIDS patients in KZN province, South Africa. The study was carried out in King Edward VIII hospital (KEH), which is a central hospital situated in Durban, South Africa. KEH provides regional and tertiary services to the whole of KZN and parts of the Eastern Cape provinces. It also serves as a teaching hospital for the University of KwaZulu-Natal medical school and also has a nursing college [31]. The study was designed as a descriptive, cross sectional quantitative survey. The study population were doctors and nurses working in different departments of the hospital such as the ARV clinic, obstetrics and gynaecology, internal medicine, surgery and paediatrics departments, and other specialised units. The questionnaire used was a standardized anonymous self-administered questionnaire with closed ended questions adapted from the "Blue Book" previously used in Tanzania by USAID for a similar study [32]. This questionnaire was designed to gather demographic information, as well as information on knowledge and comprehension of HIV/AIDS, attitude and comfort levels of HCWs towards PLWHA, and the observation of discriminatory practices [33]. Data was captured on a Microsoft Excel 2007 spread sheet and then imported to STATA version 10 [34] and SPSS version 17.0 for analysis [35]. A representative sample size was calculated using the published standardised table from the University of Florida institute of food and agricultural sciences (IFAS) [36], which gave a sample size of 333 from a population universe of 1538 HCWs at KEH with a precision level of +/-5% at 95% confidence interval (CI). Based on an assumption of a 10% non-response rate, the number of required participants was increased to compensate for this, which brought the total estimated sample size to 366, this was then rounded up to 370.

### Statistical methods

The data were summarized using descriptive statistics, with continuous variables expressed as mean or median with standard deviation and percentage for categorical variables. The independent *t *test was used to determine the relationship between knowledge and demographic variables such as gender, occupation, education, years of professional experience and designation. Knowledge is the dependent variable while demographic values are the independent variables. One way analysis of variance (ANOVA) was used if the independent variables had two or more categories such as designation, level of education and years of professional experience. Same was applied when comparing comfort and attitude (dependent variables), while demographic characteristics were the independent variables. Spearman's bivariate correlation test was used to test the strength of association between knowledge, attitude, and comfort in rendering care to PLWHA. P values less than 0.05 were considered statistically significant.

### Ethical consideration

Permission to conduct this study was granted after ethical review by the Medunsa Research Ethics Committee (MREC) of the University of Limpopo, and also from the KwaZulu Natal Department of Health and office of the CEO, King Edward VIII hospital. Informed consent was obtained from all participants after full information disclosure.

## Results

A total of 334 HCWs agreed to participate in the study, resulting in a response rate of 90.3%. Majority of participants (88.3%) were nurses of which 87.7% were female. The age range was 20 to 75 years, with a median of 37 (SD = 10.68). The age distribution of participants showed a normal curve, figure not shown. A total of 59.3% participants had at minimum of a tertiary diploma, which is equivalent to either a two or three year post-secondary education from a tertiary institution, while 13.5% had either a three or four year nursing degree, or a 5 or 6 year medical degree from a university. A further 5.1% had other postgraduate qualifications and 22% answered 'other' with regards to level of education. A quarter or 25% of the respondents worked in the medical unit or internal medicine sub-disciplines; 21% worked in surgical sub-disciplines; 14% worked in Paediatrics, 15% in obstetrics and gynaecology departments, while 25.4% worked in other departments within the hospital. Among the participants 35% had 1-5 years professional experience, 27% had 6-10 years of experience, 13.5% 11-15 years' experience, while 24% had over 15 years of experience. Other socio-demographic characteristics of participants are shown in Table 1.

**Table 1:** Demographic characteristics of participants

Characteristic	Frequency	Percent (%)
**Gender**		
Male	41	12.3
Female	293	87.7
**Educational level**		
Diploma	198	59.3
Degree	45	13.5
Postgraduate	17	5.1
Others	74	22.2
**Occupation**		
Doctor	39	11.7
Nurse	295	88.3
**Work unit**		
Medical	83	24.9
Surgical	71	21.3
Paediatrics	45	13.5
O&G	50	15.0
Others	85	25.4
**Years of experience**		
1-5 years	117	35.0
6-10 years	91	27.2
11-15 years	45	13.5
> 15 years	81	24.3

A series of 14 questions with a possible maximum score of 28 points (100%) were asked to test the knowledge of the participating HCW regarding HIV/AIDS and its transmission, since knowledge may affect the way the HCWs render service to PLWHA (Table 2). 2 points were scored for correct answers and 0 point for wrong answers or "I don't know" responses. Only 6 respondents scored less than 50%. The mean score out of a possible 28 was 20.6 (73.6%) and the median score was 20 (71.4%) (SD = 3.4). The lowest score in this category was 10 (35.7%) obtained by 0.6% (2) participants while the maximum score of 28 (100%) was obtained by 2.1% (7) participants. Tests of statistical significance between different demographic variables and knowledge of HIV/AIDS, showed that there was association between level of education and knowledge of HIV/AIDs (p ≤ 0.001); occupation and knowledge of HIV/AIDS (p ≤ 0.001); and gender and knowledge of HIV/AIDS (p = 0.004). Doctors were generally more knowledgeable about HIV/AIDs than other HCWs, probably because they are generally better educated.

**Table 2:** Responses to questions on knowledge of HIV/AIDS

Questions on Knowledge	Response Frequency (%)	
	**Correct Answer**	**Incorrect Answer**

Knowledge of different routes of HIV transmission	327 (97.9%)	7 (2.1%)
Can a mother infect her unborn baby with HIV?	325 (97.3%)	9 (2.7%)
Can HIV be transmitted by social contacts?	328 (98.2%)	6 (1.8%)
Can HIV be transmitted by sharp objects?	330 (98.8%)	4 (1.2%)
Can you identify a positive patient by looking at them?	260 (77.8%)	74 (22.2%)
Can mosquitoes transmit HIV?	306 (91.6%)	28 (8.4%)
Can the HIV virus live in the open air?	306 (91.6%)	28 (8.4%)
Do you only have to wear gloves when examining a PLWHA?	245 (73.4%)	89 (26.6%)
Risk of HIV transmission following needle prick is approx. 1 in 300	161 (48.2%)	173 (51.8%)
Risk of HIV transmission following a splash of blood to non-intact skin or mucus membrane is approx. 1 in 1,000	153 (45.8%)	181 (54.2%)
Standard sterilization procedures are sufficient when sterilizing instruments used on an HIV-positive patient	229 (68.6%)	105 (31.4%)
To prevent the transmission of HIV and other blood-borne infections in the health care setting HCW should wear latex gloves for every patient	64 (19.2%)	270 (80.8%)
Most frequent mode of contracting HIV among health workers is through work-related exposure	180 (53.9%)	154 (46.1%)
Most HIV-positive health care workers get infected at work	223 (66.8%)	111 (33.2%)

NOTE: 2 points for correct answers; 0 for incorrect answers and also for "I don't know" responses

A series of 13 different questions were asked regarding the comfort level of the HCWs when providing care to PWLHA (Table 3). Percentages and the frequency of the scores obtained from the responses were calculated to determine the individual scores on comfort in rendering care to PLWHA. 2 points were scored for answers suggestive of comfort; 0 for answers suggestive of no comfort or "I don't know" responses. The average score with regards to comfort in rendering care to PLWHA, calculated out of a maximum score of 26 marks was 16.4 (63%), with a median score of 18 (69%). The modal score on this series of questions was 26 (100%) obtained by 12.3% (41) respondents. The lowest score of zero (0) was obtained by one individual (0.3%) of respondents, while the maximum score of 100% was obtained by 12.3% (41) of respondents. Tests of statistical significance with regards to comfort level when dealing with PLWHA were calculated using the independent t test. This was only significant for gender, with males showing more comfort and empathy when dealing with PLWHA (p = 0.003)

**Table 3:** Response to questions on comfort with rendering care to PWLHA

Questions on Comfort	Response Frequency (%)		p-value
	**Positive Feeling**	**Negative Feeling**	

Comfort in giving injections to PLWHA	220 (65.9%)	114 (34.1%)	0.010
Comfortable in assisting a woman in labour with HIV or AIDS?	152 (45.5%)	182 (54.5%)	0.005
Comfortable dressing wounds of PLWHA	215 (64.4%)	119 (35.6%)	0.0001
Comfortable when conducting surgery on or suturing a person with HIV/ AIDS	149 (44.6%)	185 (55.4%)	0.030
Comfortable in setting up an IV drip for PLWHA	194 (58.1%)	140 (41.9%)	0.007
Comfortable in touching the sweat of PLWHA	227 (82.9%)	57 (17.1%)	0.0001
Comfortable in touching saliva of PLWHA	252 (75.4%)	82 (24.6%)	0.001
Comfortable when drawing blood of PLWHA	183 (54.8%)	151 (45.2%)	0.01
Are you afraid of getting HIV when caring for PLWHA	91 (27.2%)	243 (72.8%)	0.0001
Comfortable assisting or being assisted by a colleague who is HIV infected?	196 (58.7%)	138 (41.3%)	0.608
Comfortable when performing surgical or invasive procedure on clients with unknown HIV status	156 (46.7%)	178 (53.3%)	0.049
Comfortable providing health services to PLWHA	256 (76.6%)	78 (23.4%)	0.0001
Comfort in sharing bathroom with a colleague who is HIV infected	240 (71.9%)	94 (28.1%)	0.052

Note: 2 points were scored for answers suggestive of high level of comfort and 0 for answers suggestive of low levels of comfort or "I don't know" responses. P values indicate statistical significance of either predominant positive or negative feelings of comfort in rendering care to PLWHA.

With regards to attitude of HCWs to PLWHA, the mean score out of a possible 18 marks was 12.52, with a median score of 12 obtained by 26.6% (89) of respondents. 2 points were scored for answers suggestive of positive attitude; 0 for answers suggestive of negative attitude and "I don't know" responses. The lowest score of zero was obtained by one individual while the maximum score of 100% was obtained by 18% (60) respondents (Table 4). The majority of HCW had a tolerant and sympathetic attitude towards PLWHA with 92.8% (310) stating that PLWHA should not be ashamed of themselves, and another 91.3% (305) participants saying they will not be ashamed to have family members infected with HIV/AIDS. A further 85.6% (286) did not believe PLWHA should be blamed for HIV infections within the community. There was no significant difference in the levels of attitude in relation to gender, level of education and professional designation. However there was a significant relationship between years of practice (p = 0.039) and occupation (p = 0.037). The indication is that there is a significant difference in the attitude of doctors and nurses, with doctors showing a more positive attitude to PLWHA. A Spearman's correlation test was used to determine the relationship between knowledge, attitude and comfort levels to PLWHA by HCWs. There was a mildly positive correlation between knowledge and comfort levels (ρ = 0.229, p ≤ 0.001), as well as knowledge and attitude (ρ = 0.139, p = 0.011) respectively.

**Table 4:** Responses to questions on attitude

Questions on attitude	Response frequency (%)		p-value
	**Positive Attitude**	**Negative Attitude**	

Women prostitutes are responsible for the spread of HIV in our community	24 (7.2%)	182 (54.5%)	0.0001
People with HIV should not be ashamed of themselves	310 (92.8%)	24 (7.2%)	0.035
I will feel ashamed if a family member is HIV positive	305 (91.3%)	29 (8.7%)	0.784
Promiscuous men are the ones who spread HIV in our community	105 (31.4%)	229 (68.6%)	0.049
Promiscuous women are the ones who spread HIV in our community	111 (33.2%)	223 (66.8%)	0.015
HIV is a punishment from God	296 (88.6%)	38 (11.4%)	0.006
I will feel ashamed if I am infected with HIV	225 (67.4%)	109 (32.6%)	0.666
HIV is a punishment for bad behaviour	301 (90.1%)	33 (9.9%)	0.146
People with HIV/AIDS are to be blamed for bringing the disease to the community	286 (85.6%)	48 (14.4%)	0.277

Note: 2 points were scored for answers suggestive of positive attitude; 0 for answers suggestive of negative attitude and "I don't know" responses. P values indicate statistical significance of either predominant positive attitude or negative attitude towards PLWHA.

About 16% (54) respondents reported having seen patients being tested for HIV without informed consent. Another 45.8% (153) had observed or experienced patients being required to undergo HIV tests before surgery in KEH, while 38% (126) reported seeing extra precautions being used in the sterilization of medical/surgical instruments used on PLWHA. Gossiping about HIV status of patients was not uncommon amongst HCWs, with 34% (112) reportedly witnessing such incidents. The results showed that 9% (30) respondents had seen a senior healthcare practitioner diverting patients diagnosed as HIV positive to more junior or less experienced HCWs. The use of gloves for non-invasive examinations on HIV positive patients or PLWHA is also a common practice as reported by about 51% (169) of respondents (Table 5). Although 64% (215) participating HCWs were prepared to report discriminatory experiences to a responsible higher authority at the hospital. However 91% (303) reported that they have not seen PLWHA receiving less care in the hospital despite some of the discriminatory patterns of behaviour reported in this study.

**Table 5:** Responses to questions on incidence of discriminatory practices

Questions on Discriminatory practices towards PLWHA	Response frequency (%)		p-value
	Positive	Negative	
Patients being tested without consent	54 (16.2%)	280 (83.8%)	0.112
Requiring patients to be tested before surgery	153 (45.8%)	181 (54.2%)	0.032
Using latex gloves for performing non-invasive examinations on patients suspected of HIV	169 (50.6%)	165 (49.4%)	0.248
Extra precaution in sterilization of instruments used on PLWHA	126 (37.7%)	208 (62.3%)	0.079
HCW gossiping about patients HIV status	112 (33.5%)	222 (66.5%)	0.477
Senior HCW pushing HIV positive patients to junior HCW	30 (9.0%)	304 (91.0%)	0.231
PLWHA receiving less care	31 (9.3%)	303 (90.7%)	0.509
Willingness of HCW in KEH to report cases of stigmatization of PLWHA to higher authority?	215 (64.4%)	119 (35.6%)	0.132

Note: P values represent statistical significance of either predominant positive or negative responses.

## Discussion

### Knowledge of HIV/AIDs and comfort in interactions with PLWHA

The knowledge of HCWs as it relates to HIV/AIDS was moderately adequate in this study which is similar to another South African study [37], and a Nigerian study which also reported satisfactory knowledge of HIV/AIDS amongst the participating HCWs in SSA [24]. On the question, 'if mosquitoes can transmit HIV', 91.6% (306) of our participants answered correctly that mosquitoes cannot transmit HIV which is in agreement with similar reports of 94% from Nigeria [24]. In this KEH study there was a weak correlation between knowledge and comfort levels. This is reflected in the fact that HCW's who have higher levels of knowledge about HIV/AIDS and its transmission scored higher in questions about comfort levels in rendering care to PLWHA. Our results differ somewhat from the Nigerian study where HCW discomfort with taking care of PWLHA was attributed to the pervasive fear of HIV/AIDS in the community where HCWs live [24]. By contrast, a Chinese study did not show any correlation between knowledge of HIV/AIDs and comfort levels [25]. This fear of occupational exposure to HIV/AIDS was also confirmed by another South African study on rural nurses, where 58.6% of the respondents were found to be worried about occupational exposure [37]. The fear of occupational exposure to HIV/AIDS is probably one of the reasons behind the relatively low comfort levels of HCW in performing invasive surgeries on patients (53.3%) at KEH. This may be related to the perception that PLWHA deserve their illness, as they are seen as promiscuous men and women. Women are particularly stigmatized as outright prostitutes in some cases with 54.5% of participants in this study responding affirmatively to the question that "women prostitutes are responsible for the spread of HIV in our community" (p ≤ 0. 0001), and that promiscuous men and women are the ones who spread HIV within our community This observation is supported by recent reports of young girls who may travel with mini-bus taxi drivers in South Africa and are alleged to engage in transactional sex with them leading to stigmatization [38]. These findings are also similar to results from an Indian study where 44% of HCWs believed only men who patronise sex workers have to worry about HIV, and another 43% believed that sex workers are the only women who should worry about HIV/AIDS infection [27]. Though there was no significant difference in comfort levels in relation to level of education, occupational designation and years of practice experience, there was a significant relationship between gender and comfort in rendering care to PLWHA (p = 0.003), with males showing more comfort and empathy in rendering care to PLWHA. This is similar to the results reported from Nigeria [39] where male physicians where more comfortable than female physician in caring for PLWHA. This was explained by societal and cultural norms that males should be less fearful and show more bravery when compared to females [39]. Another study from Egypt [40] which attempted to determine whether a doctor's gender was predictive of HIV-related stigma and/or discriminatory practices, also found that females "gave more wrong answers" - such as believing that mosquitoes can transmit HIV - and were more likely to refuse treatment to HIV positive patients. The researchers attributed this observation to gender differences in Egyptian society as a whole, noting that women may display stronger discriminatory practices because of fear of contracting HIV, as "females would be more stigmatized than males in Egyptian society". Other cultural influences could be due to the fact that female decisions are more influenced by family ties and tradition [40]. It has been reported that women are more likely to experience HIV stigma in Africa [41], Canada, and elsewhere [42].

### Discriminatory practices by HCW towards PLWHA

In this study 45.8% (153) of the respondents said they have observed or experienced patients being required to undergo HIV tests before surgery in KEH. This is a highly contentious area because there is no perceived added advantage when patients are tested pre-operatively, since the norm is that universal precautionary measures should be taken for every patient booked for surgery. The Indian study by Mahindra et al [27] reported that 86% of HCWs agree with this action of pre-operative HIV testing. Similarly, another study from Nigeria reported that 56% of HCWs indicated that they were not prepared to operate on PLWHA [39]. A Polish study amongst doctors and nurses showed that 56% of doctors and 65% of nurses supported HIV testing of all inpatient admissions, while pre-operative testing of all surgical cases was supported by over 90% of doctors and nurses [26]. These findings and negative attitude by doctors and nurses could be traced back to the prejudicial attitudes that some HCW had towards PLWHA, associated with the belief that those infected with HIV/AIDS where promiscuous or prostitutes and deserved whatever they got. It could be also be related by fear of infection by PLWHA [24,26,39].

### Potential impacts of stigma and discriminatory practices on the management of HIV/AIDS

Stigma relates to the beliefs and attitudes of people and discrimination are the externalized stigma towards PLWHA. These are based or negative views such as prejudice, negative attitudes, abuse and maltreatment which are directed at people simply because they are seen as belonging to a particular group or are perceived as being different [11,12]. According to one authority discrimination as it is ordinarily used, refers to a process of noticing or marking a difference, often for evaluative purposes, and the most common synonyms for the verb to 'discriminate' are to 'distinguish' and to 'differentiate', which in turn denotes recognizing, discerning, appreciating or identifying difference. Concluding that "all discrimination is therefore intentional in the sense that anyone who discriminates acts on some grounds for the discrimination" [43]. Another observer argues that 'people who claims discrimination do not seek the prevention of certain irrational acts, but ask instead for the elimination in places large and small, of something like a caste system [44]. Since stigma comes in diverse forms which may include refusal to provide medical care for PLWHA, including refusal or reluctance to operate on or admit PLWHA and physical isolation in the hospital ward [45]. It can be argued that stigma may affect government efforts at curtailing the spread of HIV/AIDS [9,16,46-49] since it could affects patient's attendance at health centres for obtaining ARV and regular medical check-ups or adherence of patients to treatment plans [13-15,48]. Stigmatization also affects disclosure to family members, friends, employers, colleagues, and sexual partners [50]. Wasserstrom has suggested that the primary evil of systems of discrimination or segregation is that they "designedly and effectively marked off some persons as degraded, dirty, less than fully developed persons who were unfit for full membership in the political, social and moral community" [51]. He concludes that any system of discrimination creates a systemic disadvantage [51]. Stigma could also be due to perceived fear of occupational injuries to HCWs due to unavailability of protective equipment which will further fuel the fear of HIV infection amongst HCW. The actions of the HCWs towards patient have an impact on patient's perception of their illness and these will impact on his/ her behaviour towards receiving care for the illness [50]. One of the more surprising findings in this study is in the issue of comfort with rendering care to people with HIV/AIDS where male HCWs appeared more comfortable with rendering care to PLWHA. This is contrary to the expected instinctive nurturing character of females. This could be explained by the observation that males are expected to be braver in traditional African societies or that females are more cautious by nature in order to protect other people in their lives. Finally, the results from this study are similar to results from other studies in Africa and elsewhere, even where different methodologies and objectives were used. For example the study by Feyissa et al from Ethiopia used mixed methods of quantitative and qualitative research as well as person triangulation to study stigmatization at rural district hospitals in Southwest Ethiopia [22]. This study reported incidence of gossiping, and showed that HCW stigmatization is decreased by increased education, knowledge and training on HIV/AIDS [22], similar to results from KEH. Another study from Poland by Gañczak [26] reported fear of occupational exposure by up to 95% of nurses and doctors leading to support of mandatory testing of admitted and pre-operative patients. This percentage is much higher than the numbers from KEH, and is probably due to the fact that the cohort of HCWs in Poland are not used to dealing with PLWHA unlike the case in South Africa, where the disease is endemic. However, there was evidence that doctors with more knowledge and training on HIV/AIDs were less likely to advocate for mandatory testing for all admitted patients, similar to findings at KEH. The results at KEH were most closely related to the study by Adetoyeje et al from Nigeria [24], which was also conducted at a tertiary teaching hospital, with a similar population of doctors and HCWs as KEH. Both studies showed similarities in knowledge and attitude of HCWs in treating PLWHA, although the Nigerian study revealed that doctors had more inherent fear and less comfort when dealing with PLWHA. This KEH study was conducted at public teaching hospital in an urban area, there may be a possibility that results obtained may differ somewhat when similar studies are conducted in other settings such as rural areas or in private hospitals in South Africa.

## Conclusions

We conclude that there is some evidence of stigmatization and discrimination in the treatment of PLWHA by HCW in this tertiary hospital in KZN province, South Africa. There was overall reasonable knowledge of HIV/AIDS by HCWs, however some knowledge gaps were identified, such as the fact that 51.8% of the participants did not know the level of risk exposure to HIV infection after a needle stick injury, while 54.2% did not know that the risk involved in a blood splash accident to an intact skin. These are possible reasons why some of the HCWs are not wholly comfortable when dealing with PLWHA, and in turn harbour some negative attitudes towards patients who are HIV positive, which might affect the quality of care rendered to these patients. This study highlights the importance of continuing education among HCWs to minimize the impact of stigmatization and discrimination when dealing with PLWHA in sub-Saharan Africa. Continuing professional education and counselling for HCW will lead to better cognitive knowledge of HIV/AIDS and better coping mechanisms. Educational activities should highlight teaching on ethics and healthcare law with a clear understanding of patient's rights. Further, the incidences of gossiping may also have the effect of compromising patient confidentiality and could interfere with the doctor-patient relationship. The availability of protective gear at work will make the work place safer, thus empowering HCWs to provide quality health care services to PLHWA. It has also been suggested that HCWs should be involved in healthcare policy decision making to enable them buy-in into proposed changes, motivate HCW participation in care of PWLHA, and decrease job attrition by overworked HCWs. New forms of legislation or more rigorous enforcement of existing laws would go a long way towards minimizing the impact of discrimination and stigmatization against PLWHA. In light of recent reports of improved life-expectancy and decreased risk of HIV transmission by patients on ARVs. It is imperative that the issue of HIV related stigma should be adequately tackled so that it does not interfere with the universal uptake of ARVs, and improved management of the HIV/AIDS in KZN and other parts of South Africa.

## List of abbreviations used

AIDS: Acquired Immunodeficiency syndrome; ARV: Antiretroviral; CDC: Centers for Disease Control and Prevention, USA; CEO: Chief Executive Officer, CI: confidence interval; IFAS: Institute of Food and Agricultural Sciences; HCWs-healthcare workers; HIV: Human Immunodeficiency Virus; HRSA: Health Resource and Services Administration; KEH: King Edward Hospital, Durban; MREC: MEDUNSA Research Ethics Committee; MEDUNSA: Medical University of South Africa; IBM: International Business Machines; PLWHA: People living with HIV and AIDS; SPSS: Statistical Program for the Social Sciences; UNAIDS: United Nations Joint Programme on HIV and AIDS; USAID: United States Agency for International Development; UKZN: University of KwaZulu-Natal; USA: United States of America.

## Competing interests

The authors have indicated that they have no competing interests.

## Authors' contributions

TF participated in the conception, design and coordination of the study as well as drafting the initial manuscript. LF was involved in conceptualization of study, design and analysis of data. SCC conducted additional research on the legal aspects of stigmatization, critical analysis of manuscript for important intellectual content, and final write-up of the manuscript. All authors approved the final version of the manuscript submitted.

## Declarations

Publication of this supplement has been funded by the College of Health Sciences and the Research Office at the University of Kwazulu-Natal.

This article has been published as part of *BMC Medical Ethics *Volume 14 Supplement 1, 2013: Selected papers from the 3rd Ethics, Human Rights and Medical Law Conference (3rd EHMRL). The full contents of the supplement are available online at http://www.biomedcentral.com/bmcmedethics/supplements/14/S1.
